# Supplementary material for: Incorporation of Tumor-Free Distance and Other Alternative Ultrasound Biomarkers into a Myometrial Invasion-Based Model Better Predicts Lymph Node Metastasis in Endometrial Cancer: Evidence and Future Prospects
Source: Diagnostics (Basel). 2022 Oct 27;12(11):2604. doi: 10.3390/diagnostics12112604 (PMC9689828; doi:10.3390/diagnostics12112604)
Supplement: Supplementary file 1 [file diagnostics-12-02604-s001.zip › Supplementary material S1_Figure S1.pdf]

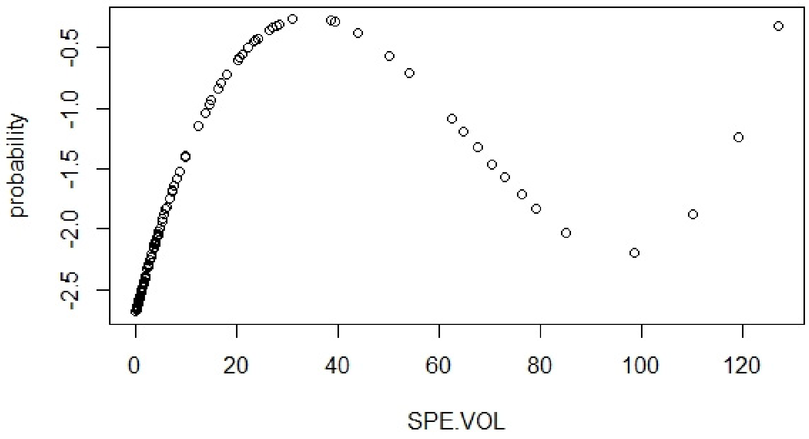

Figure S1. Tumor volume measured by ultrasound in relation to the probability of metastases to lymph nodes in the study group of 116 women with endometrial cancer.
