# Supplementary material for: Incorporation of Tumor-Free Distance and Other Alternative Ultrasound Biomarkers into a Myometrial Invasion-Based Model Better Predicts Lymph Node Metastasis in Endometrial Cancer: Evidence and Future Prospects
Source: Diagnostics (Basel). 2022 Oct 27;12(11):2604. doi: 10.3390/diagnostics12112604 (PMC9689828; doi:10.3390/diagnostics12112604)
Supplement: Supplementary file 1 [file diagnostics-12-02604-s001.zip › Supplementary material S2_Table S1.pdf]

Table S1. Univariate logit models (models D′-I) and multivariate logit model (model J) for low-risk group (n=82).

Model	Covariate	Est.	Std. Error	p-value	AIC	ACC	AUC (95% CI)	p-value (LRT)
A′′	(Intercept)	-2.71	0.50	0.000	46.39	0.768	0.605 (0.317–0.894)	0.462
	SPE-VOL	-0.01	0.02	0.422				
B′′	(Intercept)	-2.79	0.55	0.000	46.34	0.561	0.480 (0.182–0.778)	0.444
	AREA	-0.05	0.06	0.410				
C′′	(Intercept)	-1.15	0.79	0.145	43.11	0.683	0.712 (0.562–0.862)	0.051
	uTFD	-0.22	0.13	0.096				
D′′	(Intercept)	-2.53	0.73	0.000	47.13	0.829	0.625 (0.315–0.935)	0.284
	bs(SPE.VOL)1	-5.90	8.05	0.463				
	bs(SPE.VOL)2	31.17	27.0	0.248				
	bs(SPE.VOL)3	-63.13	66.4	0.342				
E′′	(Intercept)	-1.57	0.81	0.052	45.25	0.488	0.737 (0.524–0.950)	0.128
	bs(AREA)1	-12.19	7.05	0.084				
	bs(AREA)2	24.92	12.86	0.053				
	bs(AREA)3	-29.82	20.88	0.153				
F′′	(Intercept)	-4.15	2.57	0.106	52.57	0.720	0.786 (0.626–0.947)	0.104
	bs(uTFD)1	10.00	9.44	0.289				
	bs(uTFD)2	-12.25	10.95	0.263				
	bs(uTFD)3	-1.68	18.29	0.927				
G′′	(Intercept)	-3.00	0.59	0.000	44.70	0.768	0.645 (0.421–0.869)	0.135
	uMI	1.32	0.86	0.126				
H′′	(Intercept)	-3.31	0.72	0.000	43.31	0.720	0.695 (0.483–0.908)	0.057
	EMIR	1.66	0.90	0.067				
I′′	(Intercept)	-2.47	0.43	0.000	46.14	0.134	0.533 (0.505–0.561)	0.376
	uCSI	-15.10	1769.26	0.993				

J″	(Intercept)	-3.54	1.28	0.006	46.24	0.780	0.575 (0.363–0.786)	0.405
	Size	0.79	0.92	0.385				
K′′	(Intercept)	-3.11	0.59	0.000	42.27	0.854	0.691 (0.469–0.913)	
	uMI:EMIR	2.01	0.89	0.024				0.031
